# Beard hair response in alopecia areata universalis treated with baricitinib

**DOI:** 10.1016/j.jdin.2026.01.003

**Published:** 2026-01-27

**Authors:** Andrea Calogero Trecarichi, Alice Amato, Francesco Lacarrubba, Giuseppe Micali, Federica Dall’Oglio

**Affiliations:** Dermatology Clinic, University of Catania, Italy

**Keywords:** alopecia areata barbae, alopecia areata universalis, baricitinib, beard hair, JAK inhibitors, monotherapy, systemic therapy

*To the Editor:* The efficacy of baricitinib in beard alopecia areata (AA) has not been evaluated in randomized clinical trials. Only a single real-life study including 60 AA patients, of whom 14 were affected by AA universalis (AAU), investigated the use of baricitinib in combination with oral minoxidil^1^ in beard regrowth, with an overall response of 85%. We report our experience from an observational retrospective real-world single-center study on beard hair regrowth in AAU patients under baricitinib as monotherapy. The protocol was approved by the internal IRB; informed consent was individually obtained. Out of 45 AAU patients, we retrospectively identified 10 males (mean age 35.8 years: range 20-56) under baricitinib 4 mg/day for at least 32 weeks with a mean disease duration of 5.8 years (range 2-15) and mean therapy duration of 58 weeks (range 32-97). Beard status at baseline and at 4-week intervals was assessed by an adapted clinician-reported outcome (beard-ClinRO) using a grading from 0 to 3 (0 = no beard loss; 1 = minimal gaps of beard hair; 2 = evident patches of beard loss; 3 = total beard loss). Of all 10 patients that showed grade 3 beard-ClinRO at baseline, 9 responded to treatment and 1 showed no regrowth after 52 weeks. Among responders with reduction of beard-ClinRO≥1 point, 1 showed initial beard regrowth at 8 weeks, five at 12 and three at 24 weeks of treatment. In all responders, beard regrowth was observed by week 24. Five patients achieved full beard regrowth after an average of 32 weeks (Fig 1, *A* and *B*), 2 showed beard-ClinRO reduction from grade 3 to grade 1 and 2 from grade 3 to grade 2 after an average of 36 and 24 weeks, respectively. In these 4 patients no further improvement was observed so far, after an average therapy duration of 65 weeks. Overall, 90% of patients with beard AA responded to treatment. The Wilcoxon signed-rank test confirmed a statistically significant improvement from baseline (W = 45.0, *P* < .05). Five showed beard regrowth after the scalp, 2 before, and 2 in scalp nonresponders. Eyelashes and eyebrows regrowth, in most patients (44%), followed the beard (Table I). Notably, among responders, there was wide age range (20-56 years) and disease duration up to 15 years. Comparing our results to those reported by Moussa et al,^1^ patients with AAU and total beard hair loss experiencing complete regrowth were 5 out of 10 (50%) in our case series *vs* 3 out of 14 (21%) in theirs. Moreover, in our study, 4 additional patients showed partial response and are currently under follow-up. Finally, our results were achieved with baricitinib monotherapy, confirming its efficacy in the treatment of beard AA in patients affected by AAU, a subtype of AA considered the most severe and challenging to treat.^2^ Based on such results, the role of minoxidil, which is responsible for increasing terminal hair regrowth,^3^ likely promoting immunomodulation and anagen induction,^4^ when used in association with baricitinib for beard regrowth, remains, in our opinion, a matter of further investigation.

**Fig 1:**
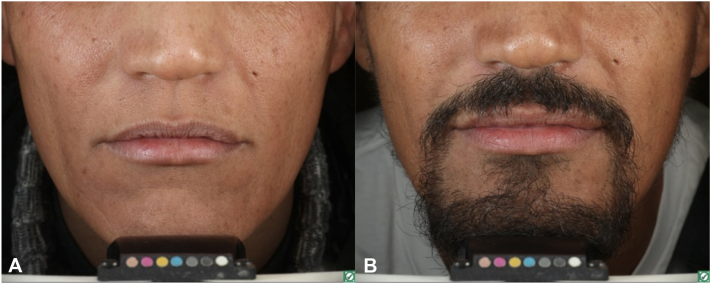
**A,** Alopecia areata of the beard in a patient affected by alopecia areata universalis; **B,** Complete beard regrowth after 32 weeks of treatment with baricitinib administered as monotherapy.

**Table I:** Patients with alopecia areata universalis under baricitinib: Demographics, clinical characteristics, outcome and timing of beard, scalp hair, and eyebrows and eyelashes regrowth

# Pt. (age)	Therapy duration (weeks)	Early responder			Gradual responder	Nonresponder	Outcome (beard ClinRO)
		1-4 weeks	5-8 weeks	9-12 weeks	13-36 weeks		
1 (35 y)	97						0
2 (39 y)	92						2
3 (26 y)	66						2
4 (52 y)	61						1
5 (20 y)	52						Nonresponder
6 (56 y)	39						0
7 (23 y)	51						0
8 (46 y)	50						0
9 (22 y)	40						1
10 (39 y)	32						0

Hair regrowth: scalp = , eyebrows = , eyelashes = , beard = .

## Conflicts of interest

None disclosed.
